# Thermodynamic Modeling of Hydrogen Storage Capacity in Mg-Na Alloys

**DOI:** 10.1155/2014/190320

**Published:** 2014-10-14

**Authors:** S. Abdessameud, M. Mezbahul-Islam, M. Medraj

**Affiliations:** Department of Mechanical and Industrial Engineering, Concordia University, 1455 de Maisonneuve Boulevard West, QC, Montreal, Canada H3G 1M8

## Abstract

Thermodynamic modeling of the H-Mg-Na system is performed for the first time in this work in order to understand the phase relationships in this system. A new thermodynamic description of the stable NaMgH_3_ hydride is performed and the thermodynamic models for the H-Mg, Mg-Na, and H-Na systems are reassessed using the modified quasichemical model for the liquid phase. The thermodynamic properties of the ternary system are estimated from the models of the binary systems and the ternary compound using CALPHAD technique. The constructed database is successfully used to reproduce the pressure-composition isotherms for MgH_2_ + 10 wt.% NaH mixtures. Also, the pressure-temperature equilibrium diagram and reaction paths for the same composition are predicted at different temperatures and pressures. Even though it is proved that H-Mg-Na does not meet the DOE hydrogen storage requirements for onboard applications, the best working temperatures and pressures to benefit from its full catalytic role are given. Also, the present database can be used for thermodynamic assessments of higher order systems.

## 1. Introduction

Hydrogen stands as an ideal fuel for the future reducing the dependence on oil and the environmental problems arising from the use of fossil fuels. Hydrogen can be used for power generation through fuel cells. Hydrogen fuel cells have a wide range of potential applications ranging from microfuel cells that power portable electronics to mobile applications [1]. The transition to hydrogen energy is hindered by technical barriers related to storage problems. Solid state hydrogen storage materials, such as lightweight metal hydrides and complex and chemical hydrides, have been widely investigated due to their small volume, low equilibrium pressure, safety advantages, and high storage capacity [2–6]. 

Magnesium and its alloys stand as promising candidates for hydrogen storage. In fact, magnesium hydride MgH_2_ contains 7.6 wt.% hydrogen [4], but it suffers from extremely slow hydriding kinetics. A temperature of about 300°C is required for a plateau pressure of 1 bar at thermodynamic equilibrium, which corresponds to an enthalpy of formation of −78 kJ/molH_2_ [4]. The investigations of magnesium hydrides found in the literature focus on decreasing desorption temperature, enhancing the kinetics and cycle life, and lowering their reactivity with air and oxygen [7–9]. Mixing with other compounds and/or incorporating new elements have been shown to be effective strategies to tune the thermodynamic properties of MgH_2_ [7, 10]. The literature shows that the search of new alloys, suitable for hydrogen storage, is somewhat a trial and error method, involving numerous experiments. Also, hydrogen is a flammable gas which makes this search more difficult. The effort and time of experiments can be reduced significantly with the application of thermodynamic calculation. Hence, a self-consistent thermodynamic database of the H-Mg-Me system (Me is one metal or more) will be very useful for identifying the most promising hydrogen storage alloys and for studying the effect of adding minor elements or mixing hydrides on the storage capability.

In recent years, the Mg-based perovskite-type hydrides, especially NaMgH_3_, have received considerable attention for hydrogen storage applications [11–17]. In addition to its high gravimetric and volumetric hydrogen densities (6 wt.% and 88 kg/m^3^), NaMgH_3_ demonstrates reversible hydrogen storage properties [18]. The crystal structure of NaMgH_3_ has been identified as orthorhombic perovskite with the space group of* Pnma* (GdFeO_3_-type structure) [15]. Recently, it has been found that NaH hydride addition greatly improves the hydrogen storage properties of MgH_2_ because of the formation of NaMgH_3_ [19]. Fast hydrogen mobility in NaMgH_3_ has been revealed by H NMR study and related to its perovskite structure [20]. Therefore, it is believed that, in a mixture of MgH_2_ and NaMgH_3_, hydrogen gas adsorption and dissociation are activated by NaMgH_3_ grains which offer fast diffusion pathway for hydrogen atoms into MgH_2 _[16, 19]. Also, it has been found that NaMgH_3_ forms during the destabilization reactions of many complex hydrides [21–27].

Consequently, accurate thermodynamic description of H-Mg-Na system is required as a building block of a larger database of H-Mg-Me. In the present work, thermodynamic modeling is used to provide a self-consistent database which can be used to predict hydrogen storage properties of the H-Mg-Na system for the whole composition range. The H-Mg-Na system is modeled using FactSage software [28].

## 2. Literature Review

### 2.1. H-Mg System

No complete experimental phase diagram of the Mg-H system could be found in the literature. An extensive literature review of the H-Mg system has been presented by San-Martin and Manchester [29] and later by Zeng et al. [30]. But some of the experimental data [31, 32] were not cited by San-Martin and Manchester [29] and are added in this work. Experimental investigations on phase equilibria were performed by different researchers [29, 33–35]. Only one temperature-composition isobar profile at 1.013 bar was predicted by Shapovalov et al. [36]. The H-Mg system consists of hcp-(Mg) (the interstitial solid solution of H in Mg) and *β*-MgH_2_ in addition to the liquid and gas phases. Two invariant equilibria have been confirmed by San-Martin and Manchester [29] in this system, L → hcp-(Mg) + gas and hcp-(Mg) + gas → MgH_2_. According to Stampfer et al. [35], from the measured pressure-composition isotherms (PCI), the composition of *β*-MgH_2_ after a complete hydriding reaction was MgH_1.99±0.01_. In this work, this phase is treated as a stoichiometric compound. The equilibrium absorption/desorption pressure of the *β*-MgH_2_ was investigated by different authors [37–42]. According to Krozer and Kasemo [41], the equilibrium formation pressure of MgH_2_ is very close to its decomposition pressure. Stampfer et al. [35] collected 129 data points in the measurement of the dissociation pressures of *β*-MgH_2_ in the temperature range 587–849 K with uncertainties of 0.35 bar and 1 K. These results are used in this work because they are self-consistent and in excellent agreement with the results published by Ellinger et al. [43], Reilly and Wiswall [44], and other groups [36, 45–47]. The enthalpy and entropy of formation of *β*-MgH_2_ have been calculated from PCI measurements using Van't Hoff plot by many researchers [35, 43–48].

Wolf et al. [31] determined the standard entropy (*S*_0_ *= *30.64 ± 0.05 JK^−1 ^mol^−1^) and the specific heat capacity function, *c*_*p*_(*T*) = (2.8711 + 0.11061*T* + 68611*T* ^−2^) JK^−1 ^mol^−1^, within the range 298–373 K for MgH_2_ using adiabatic low temperature calorimetry and differential scanning calorimetry (DSC). Bogdanović et al. [32] determined the average desorption enthalpy at an average temperature of 683 K using calorimetric measurements. They used the results published by Wolf et al. [31] to calculate the enthalpy and entropy of formation of MgH_2_. These results [31, 32] are used as first approximation in the present work together with the previously reported PCIs results.

Hydrogen solubility in magnesium has been investigated several times [34, 36, 42, 49–53]. Stampfer et al. [35] calculated the equilibrium hydrogen solubility in solid magnesium, hcp-(Mg), at five different temperatures from the PCI measurement assuming that the decomposition of MgH_2_ was complete at the end of the isotherm. A modified Sieverts apparatus was used by Koeneman and Metcalfe [42] to determine the solubility of hydrogen in magnesium between 328 and 1048 K. The results of Koeneman and Metcalfe [42] are in good agreement with those published later by Huang et al. [50] and Shapovalov et al. [34, 36]. Popovic and Piercy [53] measured the solubility of hydrogen in solid magnesium. But their [53] values are lower than those reported earlier [33, 35, 41, 49] and are not considered in the present work. The data obtained by Shapovalov et al. [36] using the conventional methods for high temperatures are also lower. Therefore these data [36] are not considered in this work because of the possible losses of hydrogen.

Thermodynamic modeling of the H-Mg system was conducted by Zeng et al. [30]. But the hydrogen solubility in the molten magnesium was neglected in their [30] work and the liquid phase was treated as an ideal solution. Recently, Harvey and Chartrand [54] modeled the hydrogen solubility in liquid magnesium using the modified quasichemical model taking into account the solubility of hydrogen in the liquid magnesium. Their optimized thermodynamic parameters for the liquid phase are used in the present work and, thus, the other phases are remodeled to be consistent with the new liquid and to take into account the new experimental data of Wolf et al. [31] and Bogdanović et al. [32].

### 2.2. H-Na System

The H-Na system was reviewed by San-Martin and Manchester [55]. Based on some of the earlier studies [56–58], they [55] predicted that at the atmospheric pressure the maximum solubility of H in solid Na should be less than 8 × 10^−5^ at.%. Since no experimental measurement of the solubility could be found in the literature their [55] prediction will be used during optimization in the present work. The solubility of H in liquid Na was measured by several researchers [59–64] in the mid-1900s in the temperature range from 373 to 723 K at the atmospheric pressure. All these measurements showed a consistent trend of increased H solubility in liquid Na with increasing temperature. These results will be compared with the present thermodynamic modeling.

The H-Na system has one stable compound, NaH. The melting point of NaH was determined by thermal analysis by Skuratov et al. [65] as 911 ± 2 K at 107.3 bar and 911 ± 2 K at 207.3 bar. Another measurement by Klostermeier and Franck [66] showed the melting point of NaH to be 905 ± 2 K at 106 bar which is in accord with Skuratov et al. [65]. The kinetic studies on the rate of reaction and thermal decomposition of NaH were carried out by [67, 68]. Prochazka and Nedved [67] studied the effect of CO on H during the reaction and suggested the CO acts as a precursor. Gwyther and Whittingham [68] measured the rate of H removal from Na + NaH mixtures by continuous evacuation and purging by argon in the temperature range of 533–693 K. They also reported the rate of H desorption from unsaturated solutions of NaH in liquid Na at ≤673 K.

Crystallographic study of the Na-H system was performed by several researchers [69–72] using X-ray diffraction. NaH has an *fcc* crystal structure ($Fm\overset{-}{3}m$) with a prototype of NaCl. The XRD measurement by Kuznetsov and Shkrabkina [72] at different temperatures did not show any phase transformation of NaH up to the decomposition temperature. They [72] also observed that the lattice parameter of NaH increases linearly from* a* = 0.4872 nm at room temperature to 0.5000 nm at 673 K. Later San-Martin and Manchester [55] reported the following equation to fit the data of Kuznetsov and Shkrabkina [72]:

(1) $$\begin{array}{c} a=0.487017+0.32{\times 10}^{-4}T\;\left(293<T<683\;\text{K}\right). \end{array}$$

Qiu et al. [73] also calculated the lattice parameter of NaH as 0.4857 nm using the first principle which is close to the reported values by [72].

The enthalpy of formation of NaH was determined by several groups using two different techniques: calorimetric methods [74–77] and dissociation pressure data [57, 65, 78]. In the calorimetric methods the enthalpy values are measured from the difference between the heat of reaction of NaH and that of Na, with water [55] as it is the most common medium due to the well-known *c*_*p*_. For the second method, the dissociation pressures of hydrides are measured usually over a temperature range. Using these data, the enthalpy of formation at the atmospheric pressure is determined from the slope of the Van't Hoff plot (log*P* versus* 1/T*) [55]. The enthalpy of formation of NaH that was reported by various groups [57, 75, 76, 78] is fairly in agreement with each other and will be compared with the present calculation. The heat capacity of NaH was measured by Sayre and Beaver [79] in the temperature range from 60 to 90 K using an adiabatic calorimeter. Their [79] reported *c*_*p*_ values will be compared with the present calculation.

A partial phase diagram of the Na-H system was presented by Predel [80]. Later Qiu et al. [73] assessed the H-Na system combining experimental data from the literature and first principle calculation based on density functional theory to supplement the thermodynamic properties of this system. Qiu et al. [73] modeled the Na-H liquid with the random solution model and bcc phase with the sublattice model. Heat capacity in the temperature range from 0 to 2000 K was calculated by first principle calculations. They [73] also presented the heat of formation of NaH, decomposition pressure, and hydrogen solubility in liquid Na.

### 2.3. Mg-Na System

The experimental work and thermodynamic modeling of the Mg-Na system were carried out by several groups [81–85]. The main feature of Mg-Na system is the large immiscibility in the liquid phase. No experimental data regarding the critical temperature as well as the shape of the immiscibility gap could be found in the literature. The solubility of Na in solid Mg as well as Mg in solid Na is negligible. Pelton [84] estimated ~0.5 at.% Na solubility in Mg by assuming Henrian behavior of the solution and employing Van't Hoff equation to back-calculate the solubility. This is rather small solubility and since there is no experimental evidence, no solubility of Na in Mg is considered in the present work.

Mathewson [81] employed thermal analysis and determined the composition and temperature of the monotectic (liquid 1 → liquid 2 + hcp-(Mg)) reaction to be 2 at.% Na and 911 K. He [81] reported the composition of liquid 2 to be ~98.6 at.% Na at 911 K. Klemm and Kunze [83] reported Mg-Na phase diagram with the monotectic temperature of 910 K. Although they [83] mentioned this as a peritectic reaction instead of monotectic, the temperature of the thermal event agreed well with the results of Mathewson [81]. Pelton [84] extracted the composition of the monotectic liquid 2 from Klemm and Kunze's [83] reported phase diagram as ~92.7 at.% Na and used this value in his assessment. The composition (~98.6 at.% Na) reported by Mathewson [81] could be associated with higher error due to the use of a glass container which usually reacts with Mg-Na liquid [84]. Hence during optimization the composition of liquuid 2 reported by Klemm and Kunze [83] will be used since they used iron crucibles. Lantratov [82] reported the temperature dependent solubility of Na in liquid Mg. The solubility of Na increases from 2.1 at.% at 911 K to 2.7 ± 0.1 at.% Na at 973 K.

Lantratov [82] measured the activities of Na and Mg in the liquid along the complete composition range by EMF method at 973 K which exhibited strong positive deviation from ideality due to the limited solubility. In a recent study, Zhang et al. [85] presented a thermodynamic model of the Mg-Na system as the constituent binary of the Al-Mg-Na ternary system. They [85] employed the random solution model for the liquid phase and calculated the Mg-Na phase diagram and the activities of Mg and Na in the liquid.

### 2.4. H-Mg-Na System

Only one ternary compound, NaMgH_3_, has been reported in the H-Mg-Na system. The standard enthalpy of formation of NaMgH_3_ was determined by Bouamrane et al. [13] as −231 ± 4 kJ/mol, by calorimetry using its reaction with diluted hydrochloric acid (0.5 M HCl). The hydrogen desorption and absorption properties of NaMgH_3_ have been investigated for the first time by Ikeda et al. [18] using XRD, thermogravimetry, differential thermal analysis, and hydrogen pressure-composition PCI measurements. They [18, 86] reported that 5.8 ± 0.2 wt.% hydrogen has been released from the sample during two endothermic reactions. Hydrogen was desorbed after only 8 min at 673 K and NaMgH_3_ formed under 10 bar of hydrogen at 673 K [18]. Only one PC isotherm at 673 K was reported [18] showing two plateau pressures of 1.5 and 0.4 bar confirming that NaMgH_3_ decomposes in two steps according to the following reactions [18]:

(2) $$\begin{array}{c} {\text{NaMgH}}_{3}⟶\text{NaH}+\text{Mg}+{\text{H}}_{2}\;4\;\text{wt}.\%\;\;{\text{H}}_{2} \end{array}$$

(3) $$\begin{array}{c} \text{NaH}⟶\text{Na}\left(\text{l}\right)+\frac{1}{2}{\text{H}}_{2}\;2\;\text{wt}.\%\;\;{\text{H}}_{2} \end{array}$$

The enthalpy change of reaction (2) was calculated by Ikeda et al. [18], using Van't Hoff plot; Δ*H* (298 K) = 88 kJ/molH_2_. Using this value and the reported standard heat of formation of NaH, −114.1 kJ/molH_2_ [87], as enthalpy change for reaction (3), the standard enthalpy of formation of NaMgH_3_ was estimated to be −96.7 kJ/molH_2 _[18] or −145 kJ/mol. PCIs of NaMgH_3_ during decomposition were reported by Komiya et al. [17] at 673, 698, and 723 K. For the isotherm measured at 673 K, the plateau pressures were around 1 and 0.4 bar for reactions (2) and (3), respectively. The enthalpy and the entropy changes of these reactions have been calculated using the Van't Hoff plot. By calculating the sum of the enthalpy changes of reactions (2) and (3), the standard enthalpy of formation of NaMgH_3_ was wrongly estimated by these authors to be −210 ± 17 kJ/molH_2 _[17]; they did not pay attention to the units used (kJ/molH_2_). Hence, heat of formation of this compound will be recalculated in the present work using their [17] enthalpy values for reactions (2) and (3). PCIs of NaMgH_3_ during decomposition were reported by Ikeda et al. [15] at 653, 673, and 693 K. Pottmaier et al. [27] investigated the thermodynamic properties of NaMgH_3_ using high pressure DSC, PCI measurements, and density functional theory calculations (DFT). Enthalpy and entropy of reaction (2) were calculated from PCI measurements at 650, 670, 680, 700, and 723 K [27]. Pottmaier et al. [27] used their results together with the experimental and calculated values from the literature to estimate the thermodynamic properties of NaMgH_3_ using the CALPHAD approach. It should be pointed out that, for the PCIs published by Ikeda et al. [15], Komiya et al. [17], and Pottmaier et al. [27], the plateaus were slopped and very limited data points were collected within them. In addition to that, the quality of the data given by Pottmaier et al. [27] is poor especially at 650 and 670 K; the plateaus are not flat with large pressure variations. Slow kinetics, an insufficient time for each equilibrium measurement, or an insufficient pressure resolution might be the causes of these problems and might have led to erroneous enthalpy and entropy determination from Van't Hoff plot. Later on, Sheppard et al. [88] investigated the kinetic and thermodynamic data of NaMgH_3_ decomposition. PCIs showing the first reaction decomposition at 671.4, 683.8, 691.9, 702.8, and 712.9 K were reported. All the above-mentioned problems have been avoided by Sheppard et al. [88] by waiting longer (more than 2 h) to reach true thermodynamic equilibrium for each sorption step. The plateau curves were wide and flat with negligible hysteresis. For all these reasons the experimental data reported by Sheppard et al. [88] are used in the present optimization of the H-Mg-Na system. The thermodynamic properties of NaMgH_3_ obtained from PCIs and DSC by different authors are summarized in Table 5. Since there is no information regarding the homogeneity range of NaMgH_3_ in the literature, this compound is treated as stoichiometric in this work. Thermodynamic modeling of the H-Mg-Na system for the whole composition range is conducted for the first time in the present study.

## 3. Thermodynamic Modeling

### 3.1. Pure Elements

The Gibbs energy functions of the pure elements (Mg, Na) are taken from the SGTE (Scientific Group Thermo data Europe) compilation of Dinsdale [89]. These data are taken in reference to the Stable Element Reference* (SER)* at 298.15 K and 1 bar. Liquid monoatomic hydrogen is not stable under normal conditions; its Gibbs energy has been estimated by Roy and Rodgers [56] and is reported in Table 1. The *c*_*p*_ values of the gases included in this study, that is, H_2_, H, Mg, Mg_2_, MgH, Na, Na_2_, and NaH, are taken from* NIST-JANAF* thermochemical tables [90] compiled by FactPS database [28].

### 3.2. Stoichiometric Compounds

The Gibbs energy of a binary stoichiometric phase is given by

(4) Gϕ=xiGiϕ10+xjGiϕ20+ΔGf,

where *x*_*i*_ and *x*_*j*_ are mole fractions of the components *i* and *j* of the compound denoted by *f*. ^0^ *G*_*i*_^*ϕ*_1_^ and ^0^ *G*_*i*_^*ϕ*_2_^ are the Gibbs energy of components *i* and *j* in their standard state. Δ*G*_*f*_ = *a* + *bT* is the Gibbs energy of formation per mole of atoms of the stoichiometric compound. The parameters *a* and *b* are obtained by optimization. The stoichiometric compounds in the H-Mg-Na system are MgH_2_, NaH, and NaMgH_3_.

### 3.3. Liquid Phase

Modified quasichemical model is used to describe the liquid phase for all the binaries. This model uses the energy of pair formation to define the excess Gibbs energy. According to [91], the excess energy is expressed as

(5) $$\begin{array}{c} \Delta g_{AB}=\Delta g_{AB}^{0}+\sum_{i\geq 1}g_{AB}^{i0}X_{AA}^{i}+\sum_{j\geq 1}g_{AB}^{0j}X_{BB}^{j}, \end{array}$$

where Δ*g*_*AB*_^0^, Δ*g*_*AB*_^*i*0^, and Δ*g*_*AB*_^0*j*^ are the parameters of the model and are expressed as functions of temperature (Δ*g*_*AB*_^0^ = *a* + *bT*). The short range ordering in the liquid is expressed by the atom to atom coordination number “*Z*” and is given by

(6) $$\begin{array}{c} \frac{1}{Z_{A}}=\frac{1}{Z_{AA}^{A}}\left(\frac{2n_{AA}}{2n_{AA}+n_{AB}}\right)+\frac{1}{Z_{AB}^{A}}\left(\frac{n_{AB}}{2n_{AA}+n_{AB}}\right),\\ \frac{1}{Z_{B}}=\frac{1}{Z_{BB}^{B}}\left(\frac{2n_{BB}}{2n_{BB}+n_{AB}}\right)+\frac{1}{Z_{BA}^{B}}\left(\frac{n_{AB}}{2n_{BB}+n_{AB}}\right). \end{array}$$

*Z*_*AA*_^*A*^ and *Z*_*AB*_^*A*^ are the values of *Z*_*A*_ when all the nearest neighbors of *A* atom are *A*'s and when all the nearest neighbors of *A* atom are *B*'s, respectively. The same applies to *Z*_*BB*_^*B*^ and *Z*_*BA*_^*B*^. All binary liquid thermodynamic parameters have been interpolated using the asymmetric Kohler-Toop technique [91]. According to Qiao et al. [92], H is singled out as the asymmetric component since Mg-Na system shows significantly different thermodynamic characteristics than both Mg-H and Na-H. In the current work, no ternary parameters are added to the liquid model.

### 3.4. Gas Phase

In the pressure range of interest, the nonideal contribution of pressure to the Gibbs energy for the gases is very small. Therefore, the gases included in this work are taken as ideal gases. The gas phase is described by the ideal solution model as

(7) $$\begin{array}{c} G=x_{i}G_{i}^{\phi }+x_{j}G_{j}^{\phi }+RT\left[x_{i}\ln x_{i}+x_{j}\ln x_{j}\right], \end{array}$$

where *i* and *j* are the gas constituents, *G*_*i*_^*ϕ*^ = ^0^ *G*_*i*_^*ϕ*^ + *RTLnP*, and *P* is the pressure.

### 3.5. Solid Solution Phases

Hydrogen atoms occupy interstitial positions in the solid magnesium, hcp-(Mg), and sodium, bcc-(Na). These phases are described by a two-sublattice model where the first sublattice is occupied by the metal atoms and the second one by hydrogen atoms and vacancies, (M)_*a*_(H,Va)_*c*_. The Gibbs energy is described by the equations:

(8) G  =  Gref+Gideal+Gexcess,Gref=∑yilyjm⋯ykqG(i:j·…·k)0,Gideal=RT∑lfl∑iyilln⁡yil,Gexcess=∑yilyjlykm∑γ=0Lγ(i,j):k×(yil−yjl)γ,

where *i*, *j*, …, *k* are components or vacancy and *l*, *m*, and *q* represent sublattices.*y*_*i*_^*l*^ is the site fraction of component *i* on sublattice *l*. *f*_*l*_ is the fraction of sublattice *l* relative to the total lattice sites. ^0^ *G*_(*i*:*j*·…·*k*)_ represents a real or a hypothetical compound energy. ^*γ*^ *L*_*(i,j)*_ represent the interaction parameters which describe the interaction within the sublattice. According to Frisk [93] the number of sites on each sublattice (M)_*a*_(H,Va)_*c*_ is *a* = 2 and *c* = 1 for the hcp-(Mg) phase and *a* = 1 and *c* = 3 for bcc-(Na) phase. To allow for the solubility of Na in hcp-(Mg) and Mg in bcc-(Na), Mg and Na are allowed to mix randomly in the first sublattice. Therefore, the hcp-(Mg) phase and bcc-(Na) in the ternary system are described by the two sublattices (Mg,Na)_2_(H,Va)_1_ and (Na,Mg)_1_(H,Va)_3_, respectively.

## 4. Results and Discussion

The thermodynamic parameters optimized in the present work for the H-Mg-Na system are given in Table 1.

### 4.1. H-Mg System

The optimized thermodynamic parameters obtained by Harvey and Chartrand [54] using the MQM for the liquid phase are used in the present work. The hcp-(Mg) is modeled using (Mg)_2_(H,Va)_1_ two-sublattice model [93] as discussed in Section 3.5. No excess terms have been employed to represent hcp-(Mg). MgH_2_ is considered a stoichiometric compound. The gas species H, H_2_, Mg, Mg_2_, and MgH are treated as ideal gases. All the parameters for this system are listed in Table 1.

**Figure 1:**
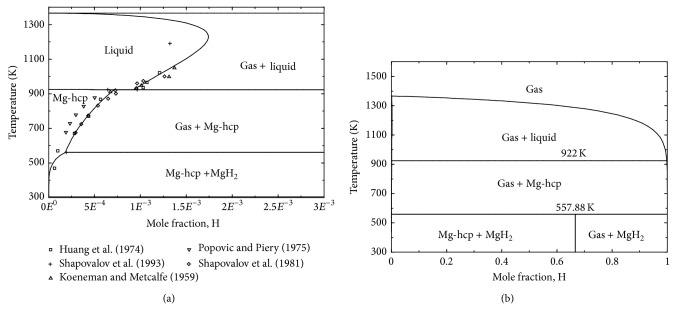
Calculated Mg-rich part of Mg-H phase diagram at 1 bar compared with experimental hydrogen solubilities in solid magnesium data from literature (a) and the calculated Mg-H phase diagram over the entire temperature range (b) at 1 bar.

The calculated enthalpy and entropy of formation of magnesium hydride MgH_2_ are given in Table 2 together with experimental data from the literature. Very good consistency can be seen between the calculated values and the experimental data [31, 34, 37, 42–47, 93] except for some deviation from the results published by Selvam et al. [38], Ellinger et al. [43], and Pedersen et al. [48]. Considering the consistency among the other six works, this deviation can be related to the quality of the PCIs and the slow kinetics which can lead to erroneous values of the equilibrium pressures. The calculated Mg-rich part of the Mg-H phase diagram at 1 bar is presented in Figure 1(a) compared with experimental hydrogen solubility data in solid magnesium. The entire phase diagram at 1 bar is shown in Figure 1(b). It can be seen in Figure 1(a) that there is a good agreement between the calculated phase diagram at 1 bar and the selected experimental data except for the results of Popovic and Piercy [53] who differed from otherwise consistent results of [33, 35, 41, 49]. According to the present calculations, the eutectic type reaction L → hcp-(Mg) + gas occurs at 0.0923 at.% hydrogen and 922 K which agrees well with that calculated by Zeng et al. (0.093 at.% H and 922.8 K) [30]. There is no measured decomposition temperature for MgH_2_ at the atmospheric pressure. However, the present work predicts that MgH_2_ decomposes to hcp-(Mg) and H_2_ at 557.88 K; this value is about 3 K lower than that predicted by Zeng et al. [30].

**Figure 2:**
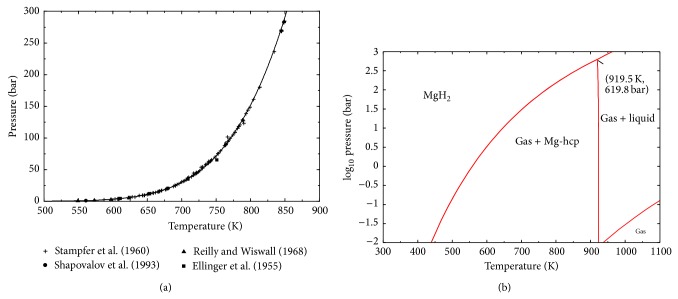
(a) The dissociation pressure of MgH_2_ calculated in this work compared to experimental data. (b) Predicted pressure-temperature diagram of MgH_2_.

The calculated dissociation pressure of MgH_2_ as function of temperature is presented in Figure 2(a) which shows good agreement with the experimental data from the literature. The present results show that the thermodynamic functions used in this work describe the H-Mg system in a broader pressure range more accurately than those reported by Zeng et al. [30]. In fact, Zeng et al. [30] unlike the current work did not achieve agreement with the experimental results above 150 bar. They blamed this inconsistency on the experimental data. The pressure-temperature diagram of MgH_2_ is presented in Figure 2(b) to show the stability of the different phases. It is predicted that MgH_2_ decomposes directly to liquid and gas above 919.5 K and 618.8 bar.

**Figure 3:**
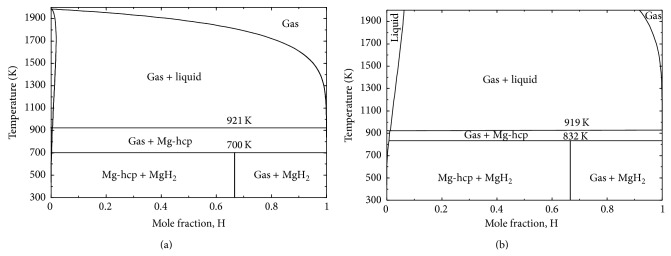
Calculated Mg-H phase diagram at (a) 30.48 bar and (b) 236 bar.

In order to compare with the experimental data of [35, 43], the H-Mg phase diagrams at 30.48 bar and 236 bar are also calculated in the present work as shown in Figures 3(a) and 3(b), respectively. The dissociation temperature of MgH_2_ is predicted to be 700 K at 30.48 bar and 832 K at 236 bar which agrees very well with the values reported by Stampfer et al. [35] (700 K at 30.48 bar) and by Ellinger et al. [43] (834 K at 236 bar). It can be seen in Figure 3 that the temperature of the reaction L → hcp-(Mg) + gas does not change a lot with pressure.

### 4.2. H-Na System

The liquid phase of the H-Na system is modeled using the modified quasichemical model. The solution has been considered to be random with no preferential short range ordering. The parameters of the model are determined considering the experimental data of H solubility in liquid Na. The bcc-(Na) is modeled using the compound energy formalism employing (Na)_1_(H,Va)_3_ two-sublattice model [93] as mentioned in Section 3.5. This model has been adopted from Qiu et al. [73]. Two excess terms are used to describe this phase. NaH is considered a stoichiometric compound. The “*c*_*p*_” ranges (0 < *T* < 298.15 K and 298.15 < *T* < 2000 K) of solid NaH have been optimized in this work to comply with the experimental data of the phase diagram as well as the thermodynamic properties. The gas species H, H_2_, Na, Na_2_, and NaH are treated as ideal gases. All the parameters for this system are listed in Table 1.

**Figure 4:**
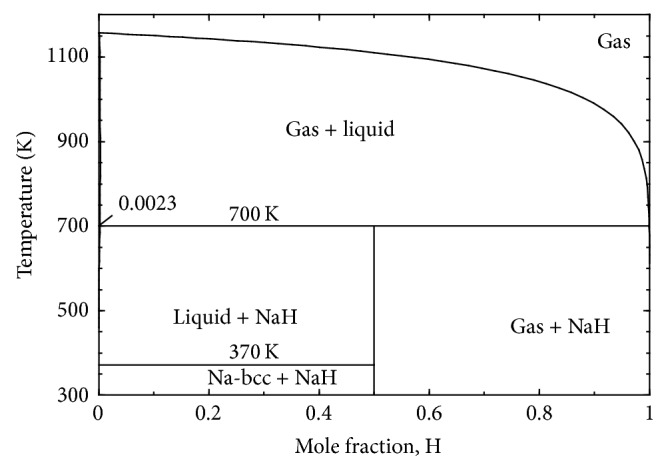
Calculated H-Na phase diagram at 1 bar.

The calculated phase diagram of H-Na system at 1 bar is presented in Figure 4. There is only one intermediate compound NaH in the system which decomposes at 700 K to liquid and gas. The H solubility in liquid Na has been found to be ~0.23 at.% at 700 K which is in good agreement with the proposed solubility limit of ~0.2 at.% by San-Martin and Manchester [55].

**Figure 5:**
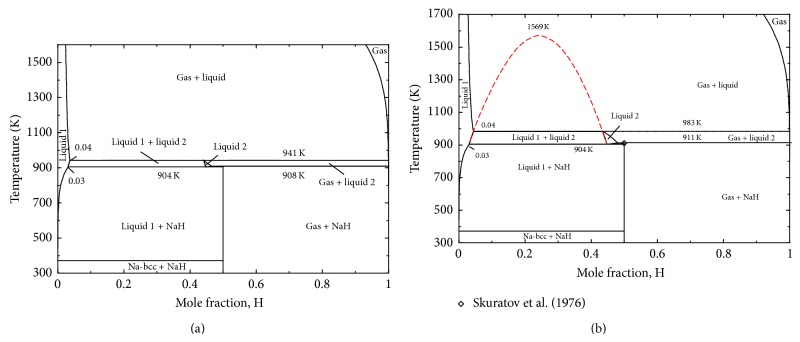
The Na-H phase diagram calculated at (a) 150 bar and (b) 200 bar showing the metastable immiscibility gap.

In order to visualize the impact of pressure on the H solubility in liquid Na, the Na-H phase diagram has been calculated at 150 bar and 200 bar as shown in Figures 5(a) and 5(b). It can be seen that the solubility increases from ~0.23 at.% (700 K) to ~4.0 at.% (983 K) from the ambient pressure to the 200 bar. The melting temperature of NaH is 911 K and 905 K at 200 and 115 bar, respectively, in the present work. This is in agreement with the measured value of 911 ± 2 K at 207.3 bar by Skuratov et al. [65] and 905 ± 2 K at 106 bar by Klostermeier and Franck [66]. The critical temperature (*T*_*c*_) for the immiscibility in the liquid is 1569 K in the present calculation as shown by the dotted line in Figure 5(b) which agrees with the 1500 ± 70 K estimated by Klostermeier and Franck [66].

**Figure 6:**
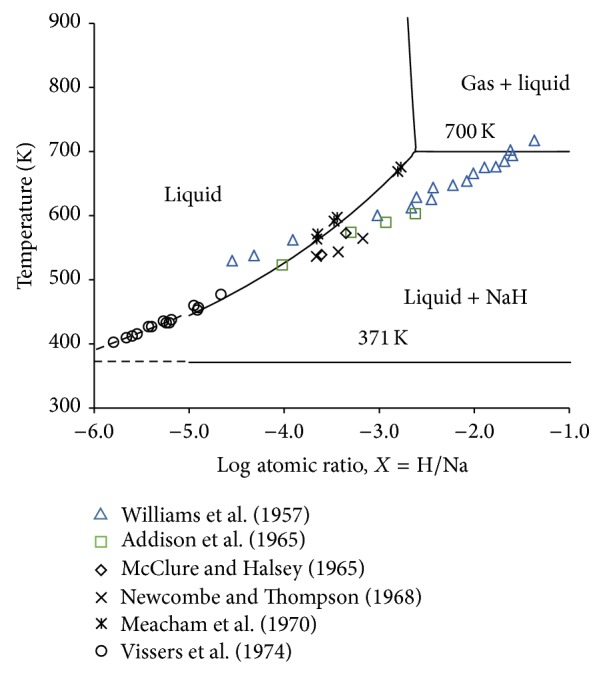
Calculated hydrogen solubility in the liquid Na system at 1-bar pressure in comparison to the experimental data in the literature [59–64].

Several measurements [59–64] on the H solubility in liquid Na were found in the literature. The solubility values are very small. Hence to compare the experimental data with the present calculation a phase diagram of temperature versus log H/Na is plotted in Figure 6. This diagram shows the Na-rich side of the phase diagram from 371 to 900 K. The solubility values are in general agreement with those from the literature. The cutoff point of the calculation in FactSage program is 1 × 10^−5^  . Therefore dotted lines have been used in the figure to extend parting lines of the phases. Some of the experimental solubility data [59, 60, 62] do not agree well with the present calculation. However, San-Martin and Manchester [56] noted that these measurements suffered from contamination due to the reaction of Na with glass walls. Therefore, during optimization only the other experimental data reported by Meacham et al. [63] and Vissers et al. [64] were given a higher weight in the present work.

**Figure 7:**
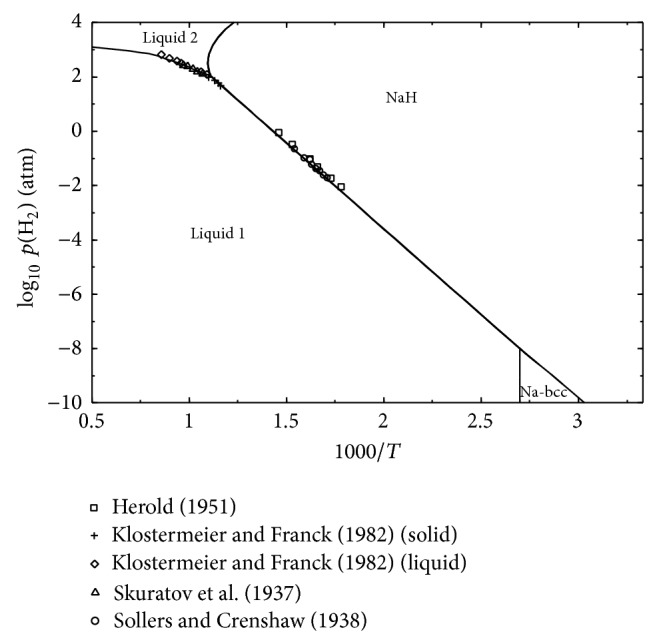
Calculated dissociation pressure of NaH in comparison with the experimental data from the literature.

The chemical potential diagram for the H-Na system calculated in the present study is shown in Figure 7. The diagram shows reasonable agreement with the experimental data from the literature [57, 58, 65, 66]. It can be seen from this diagram that, with increasing pressure, the dissociation temperature of NaH increases until ~114 bar (at 911 K) where melting of this compound occurs. After this point the slope of the curve decreases as no more NaH can dissolve in the liquid. This indicates the immiscibility of the two liquids. Similar observations were also reported by Qiu et al. [73].

**Figure 8:**
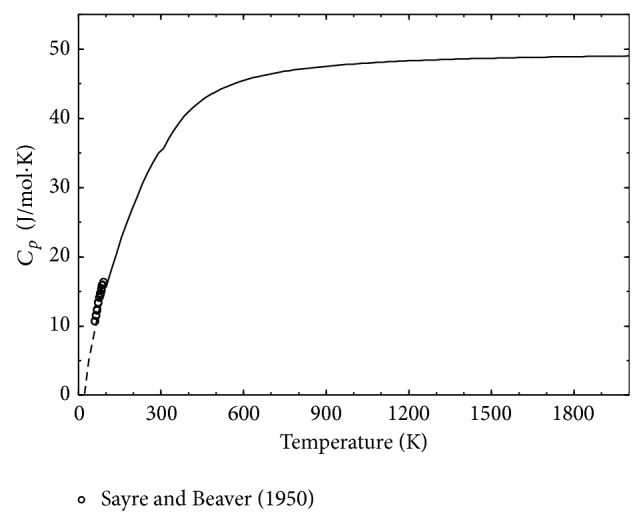
Calculated heat capacity *c*_*p*_ of NaH in comparison with experimental data [79].

The enthalpy of formation of the solid NaH has been determined as −56.98 kJ/mol, which is consistent with the available experimental data as can be seen in Table 3. The calculated heat capacity of solid NaH with the available experimental data from Sayre and Beaver [79] is shown in Figure 8. In order to obtain reliable agreement with experimental data, the Gibbs energy of the solid NaH in the temperature range 0 < *T* < 298.15 K has been determined in this work. The Gibbs energy of this compound at higher temperatures (298.15 < *T* < 2000 K) is taken from Qiu et al. [73].

### 4.3. Mg-Na System

**Figure 9:**
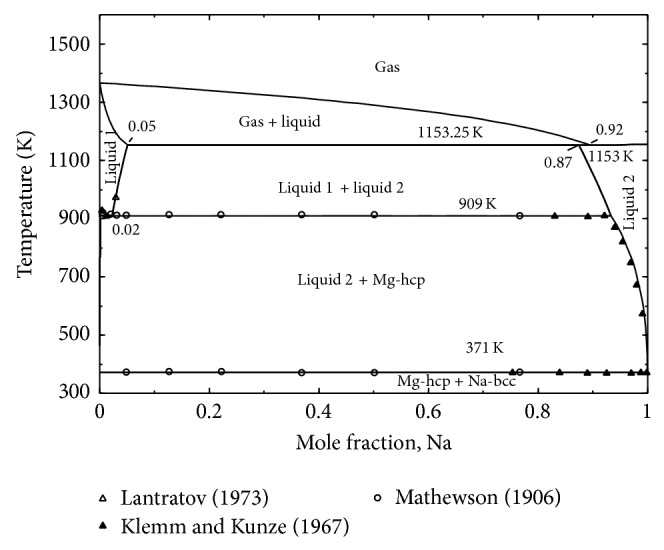
The calculated phase diagram for Mg-Na system in comparison with the experimental Data.

**Figure 10:**
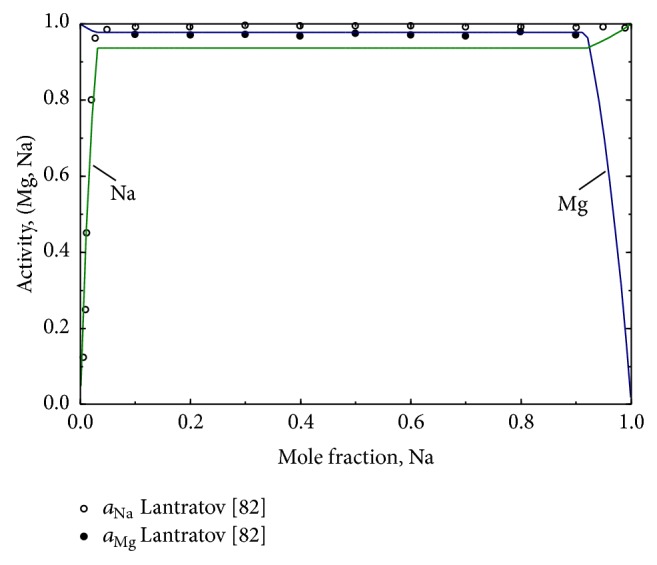
The calculated activities of liquid Na and liquid Mg at 973 K in comparison with the experimental data [82].

The calculated phase diagram of the Mg-Na system is presented in Figure 9. The thermodynamic model parameters obtained for the system are given in Table 1. The calculated phase diagram is in good agreement with the experimental data form the literature [81–85]. However, it differs a little from the calculation of Zhang et al. [85] at high temperature when the gas phase interacts with the liquid immiscibility gap. Zhang et al. [85] reported the gas→liquid 2 transformation at 97.01 at.% Na which was found at 92.5 at.% Na in the present work. Since there is no experimental data for this reaction the present prediction is acceptable. The composition and temperature of the invariant reactions in the Mg-Na system calculated in the present work are compared with the experimental data from the literature, Table 4. Activities of Mg and Na in the liquid at 973 K are calculated and presented in Figure 10. The calculated Mg activity is in good agreement with the experimental measurements by Lantratov [82]. The Na activity shows deviation from that of Lantratov [82] in the liquid immiscibility gap. However, the experimental data showed unrealistic activity almost equal to unity and was not possible to obtain without deviating from the experimental phase diagram.

### 4.4. H-Mg-Na System

Calculated changes in enthalpy and entropy for reactions (2) and (3) and enthalpy and entropy of formation of NaMgH_3_ are given in Table 5 in comparison with experimental data from the literature. There is a good consistency between the calculated heat of formation of NaMgH_3_ in this work and the experimental values reported in the literature [15, 17, 18, 89] except for the DSC results published by Bouamrane et al. [13] which are higher. This can be attributed to the use of DSC for the measurement of thermodynamic properties of the hydrides instead of PCI method which is more accurate due to its reliance on the change in volume. The calculated enthalpy and entropy of reactions (2) and (3) are in very good agreement with the values reported by Sheppard et al. [88]. Agreement is also shown between the calculated enthalpy of reaction (2) with Ikeda et al. [18] as well as between the calculated entropy of reaction (3) and Komiya et al. [17]. As discussed in Section 2.4, all the other differences are related to the PCIs quality and poor kinetics in [15, 17, 27].

**Figure 11:**
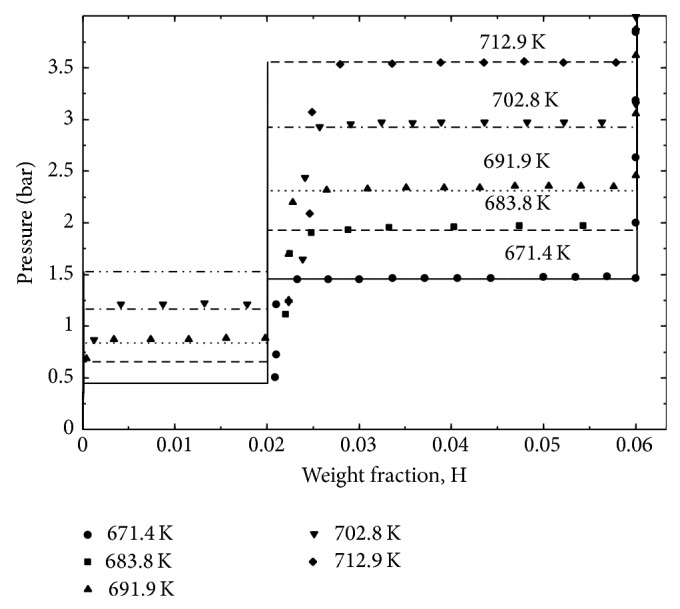
Calculated PCIs for NaMgH_3_ at various temperatures compared to experimental data reported by Sheppard et al. [88].

The calculated PCI profiles at various temperatures are presented in Figure 11 in comparison with the experimental data reported by Sheppard et al. [88]. It has been reported by these authors [88] that the samples used for the PCI measurements were composed of 84.3 wt.% NaMgH_3_, 4.7 wt.% NaH, and 11.0 wt.% MgO. In the present work, the hydrogen wt.% desorbed from the samples has been recalculated to consider only the NaMgH_3_ content assuming no reaction between H_2_, MgO, and NaH. The hydrogen content of the samples was calculated by subtracting the hydrogen desorbed from the total hydrogen content before desorption (6 wt.%). These results (Figure 11) show that the model used to describe the NaMgH_3_ reproduces the equilibrium pressures at different temperatures. The calculated PCI at 671.4 K in comparison with the experimental one shows that there is agreement between the theoretical and the measured hydrogen content of the first sample. It can be seen that the amount of hydrogen desorbed from the samples is decreasing after each experiment probably because of incomplete hydriding or dehydriding reactions and very sluggish kinetics.

**Figure 12:**
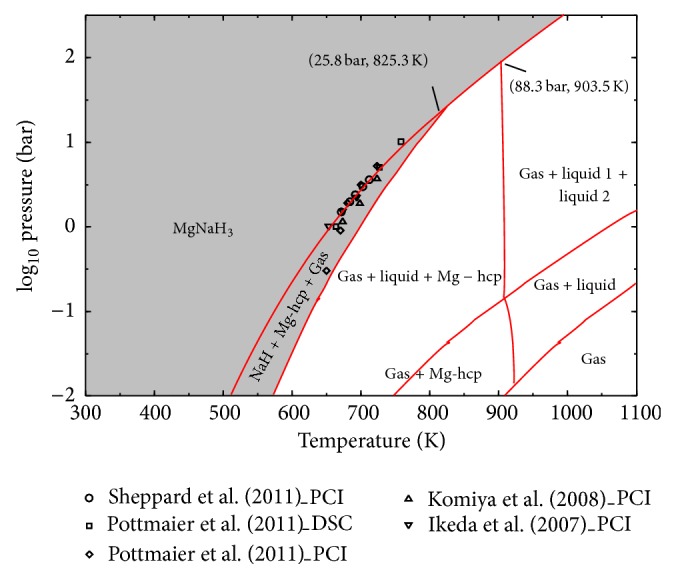
Calculated pressure-temperature equilibrium diagram for NaMgH_3_ in comparison with experimental data from the literature.

The calculated pressure-temperature diagram of NaMgH_3_ is presented in Figure 12 in relation to experimental data from the literature. There is good agreement between the calculated and the experimental data except for the PCI results obtained by Pottmaier et al. [27] at 650 K. This deviation was expected because of the poor quality of the PCIs reported by Pottmaier et al. [27] due to very low kinetics especially for low temperature experiments as discussed before. Figure 12 indicates that NaMgH_3_ is stable up to higher temperatures when the pressure is increased (up to ~900 K at ~100 bar). However, the stability region for NaH is relatively small. It can be seen in Figure 12 that the two-step decomposition of NaMgH_3_ through reactions (2) and (3) transforms into a single step decomposition (NaMgH_3_ → liquid + hcp-(Mg) + gas) from 825.3 K and 25.8 bar.

Pottmaier et al. [27] observed an important decrease in hydrogen capacity of NaMgH_3_ after 15 cycles of hydrogenation/dehydrogenation and observed at the end of the measurements that metallic Na segregated into isolated blocks which, according to these authors [27], caused this loss in capacity. Sheppard et al. [88] reported a dramatic reduction in the rehydrogenation reaction kinetics of the products (Na (l) and hcp-(Mg)) to form NaMgH_3_ and supported Pottmaier et al. [27] conclusion. This suggests that avoiding liquid Na during the decomposition of NaMgH_3_ significantly improves the kinetics and prevents capacity degradation. According to the present calculation the best working temperatures and pressures for NaMgH_3_ are shown as the shaded region in Figure 12 as this region avoids the liquid formation.

**Figure 13:**
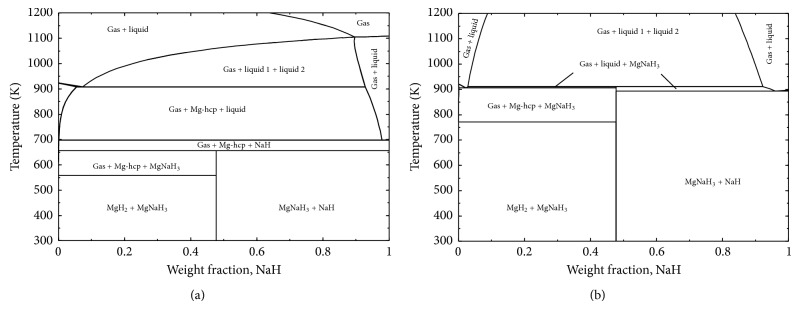
Calculated vertical section of Mg-Na-H system along the composition line MgH_2_-NaH at (a) 1 bar and (b) 100 bar.

It has been shown by Wang et al. [19] that the addition of 10 wt.% of NaH greatly improved the hydrogen storage properties and the hydrolysis properties of MgH_2 _due to the formation of NaMgH_3_. As mentioned earlier, the perovskite structure allows fast hydrogen mobility and gives NaMgH_3_ the catalytic role necessary for hydrogen storage capacity improvement of MgH_2_. In Figure 13, the calculated vertical section of Mg-Na-H system along the composition line MgH_2_-NaH at 1 bar and 100 bar is presented. Figure 13 shows that when 10 wt.% of NaH is added to MgH_2_, NaMgH_3_ forms. Also, all phase transformations with temperature at 1 bar and at 100 bar can be inferred from this figure. Since this figure does not provide the relative amounts of each phase, phase assemblage diagrams are calculated as will be discussed below.

**Figure 14:**
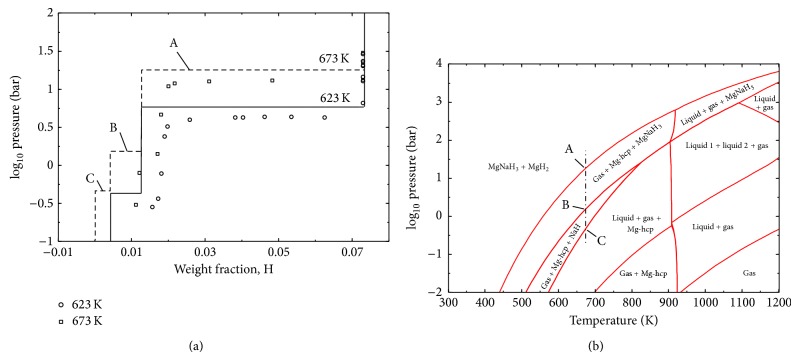
(a) Calculated P-C isotherm for MgH_2_ + 10 wt.% NaH at 623 and 673 K compared to experimental data [19]. (b) Calculated pressure-temperature diagram for MgH_2_ + 10 wt. % NaH.

Calculated PCI curves for the MgH_2_ + 10 wt.% NaH mixtures at 623 and 673 K are shown in Figure 14(a) in comparison with the results published by Wang et al. [19]. The hydrogen content of the samples in [19] has been recalculated assuming that the initial samples' hydrogen capacity is 7.3 wt.% based on the chemical formulae assuming stoichiometric amounts. At 673 K, three plateau pressures are shown in the calculated PCI curve (see arrows A, B, and C in Figure 14). The higher plateau (A) corresponds to the decomposition of MgH_2_; the second (B) and the third (C) plateaus correspond to the decomposition of NaMgH_3_ through reactions (2) and (3), respectively. At 623 K, only the first two plateaus are observed and the formation of liquid Na through reaction (3) is avoided. The last plateau (C in the PCI curve at 673 K) and the two last plateaus (in the PCI curve at 623 K) could not be observed by Wang et al. [19] and their reported plateau pressures are slightly lower than the current calculations. No information has been given by Wang et al. [19] about their PCI measurements conditions, but very few data points are shown in the published curves. It has been pointed out by Wang et al. [19] that their testing temperatures for kinetic measurements have been chosen below 623 K to avoid the decomposition of NaMgH_3_, but, according to the present calculations, NaMgH_3_ decomposes at this temperature through reaction (2) and only reaction (3) is avoided during desorption in the pressure range of measurement.

The calculated pressure-temperature diagram for the MgH_2_ + 10 wt.% NaH composition is shown in Figure 14(b). The plateau pressures at 673 K shown in Figure 14(a) (A, B, and C) are also indicated by arrows in Figure 14(b). It can be seen that Figure 14(b) is a superimposition of Figure 2(b) (pressure-temperature diagram for MgH_2_) and Figure 12 (pressure-temperature diagram for NaMgH_3_) for temperatures below ~900 K. This result suggests that the thermodynamic properties of MgH_2_ and NaMgH_3_ are not affected when mixing MgH_2_ with NaH. Only the amount of hydrogen desorbed from the mixture (the plateaus width) at each step changes with the amount of NaH added. In other words, from a thermodynamic point of view, addition of NaH to MgH_2_ does not improve the hydrogen storage properties of MgH_2_. On the contrary, the absorption/desorption kinetics is significantly improved as reported in the literature. For this purpose as well, the full potential of the catalytic role of NaMgH_3_ can only be obtained if the processing conditions are set as shown by the shaded region in Figure 12.

The decomposition temperature and the amount of released hydrogen for any compositions of the H-Mg-Na system can be obtained at any pressure using the current database. The calculations for three different pressures (1, 0.1, and 1 × 10^−4^ bar) are discussed below.

**Figure 15:**
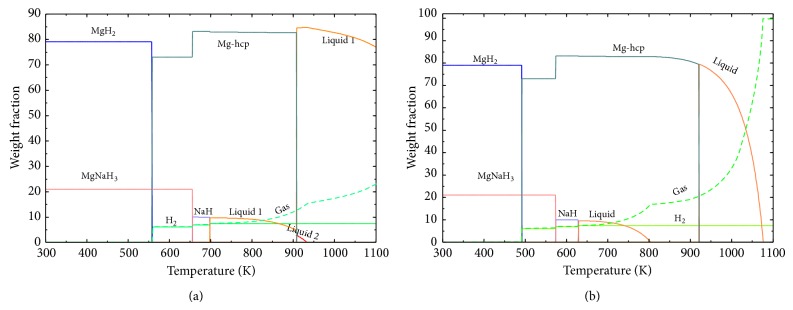
Calculated reaction path for MgH_2_ + 10 wt.% NaH at (a) 1 bar and (b) 0.1 bar.

The calculated reaction path of the MgH_2_ + 10 wt.% NaH at 1 and 0.1 bar is given in Figures 15(a) and 15(b), respectively. The reaction path at 1 bar is as follows: MgH_2_ decomposes first at 557.8 K to hcp-(Mg) and 6 wt.% H_2_ gas. NaMgH_3_ decomposes second at 656 K to NaH, hcp-(Mg), and an additional 0.9 wt.% H_2_ gas. At 697 K, NaH decomposes to liquid and an additional 0.4 wt.% H_2_ gas. Slow decrease in the amount of liquid phase from 700 K is accompanied with the evaporation of Na. The total amount of gas phase is presented in dashed line. A second liquid phase appears at the melting point of magnesium and disappears at 933 K where Mg starts to evaporate with decreasing the amount of the liquid phase. At 0.1 bar, as expected, the decomposition temperatures of the hydrides, the evaporation points of Na and Mg, and the sublimation point of Mg are lower. MgH_2_ decomposes at 491 K to hcp-(Mg) and 6 wt.% H_2_ gas and NaMgH_3_ at 573 K liberating an additional 0.9 wt.% H_2_ gas. NaH decomposes at 628 K to liquid and an additional 0.4 wt.% H_2_ gas. According to the present calculation, at a pressure of 1 × 10^−4^ bar, MgH_2_ decomposes at 366 K, NaMgH_3_ at 418 K, and NaH at 486 K. It is concluded in this work that MgH_2_ will decompose at temperatures lower than 373 K with good kinetics at low pressures if it is mixed with a small amount of NaMgH_3_.

## 5. Conclusion

A self-consistent thermodynamic database has been constructed to describe the H-Mg-Na system. Thermodynamic modeling of the constituent binary systems, H-Mg, H-Na, and Mg-Na, has been carried out. The modified quasichemical model is used to describe the liquid phase. Thermodynamic calculations of various phase diagrams and thermodynamic properties are compared with the experimental data and found to be in good agreement. The binary thermodynamic parameters of the liquid phases were interpolated using the asymmetric Kohler-Toop technique. The solid solution phases, hcp-Mg and bcc-(Na), are described by two-sublattice models as (Mg,Na)_2_(H,Va)_1_ and (Na,Mg)_1_(H,Va)_3_. The constructed database is used to predict the PCIs and the pressure-temperature diagram of the MgH_2_ + 10 wt.% NaH mixture. The calculations provide more insight into the reactions when compared to experimental data from the literature. The reaction path of the MgH_2_ + 10 wt.% NaH mixture is predicted at different pressures. In this study, it is demonstrated that H-Mg-Na system does not satisfy all the DOE requirements for onboard hydrogen storage applications (desorption temperature < 373 K, at atmospheric pressure) but still is very good candidate for high temperature applications. According to this work, at 1 bar, NaMgH_3_ decomposes at 656 K to NaH and hcp-(Mg) liberating 6 wt.% of hydrogen gas. The present database is used to find the best working temperatures and pressures of NaMgH_3_ to avoid its full decomposition and benefit from its full catalytic role when mixed with MgH_2_. At pressures of 1, 0.1, and 10^−4^ bar, the limiting working temperature should be 697, 628, and 486 K, respectively. It is also found that MgH_2_ decomposes at 366 K at a pressure of 10^−4^ bar. The present database can be used for further thermodynamic assessments of higher order systems.

## Figures and Tables

**Table 1:** Optimized model parameters for the different phases in the H-Mg-Na system (J/mole).

Phase	Model	Parameters
Liquid	MQM	*g*_H(l)_^0^ = 74,266.7 − 26.2456*T* + 20.7856*T*ln⁡*T* [54]
		*Z*_MgH_^Mg^ = *Z*_MgH_^H^ = 6, Δ*g*_MgH_^0^ = −18,049.78 [54]
		*Z*_NaH_^Na^ = *Z*_NaH_^H^ = 6, Δ*g*_NaH_^0^ = −39,245.92 + 8.45*T*
		Δ*g*_NaH_^10^ = 12,133.6 − 0.711*T*
		Δ*g*_NaH_^01^ = −66,944 + 8.368*T*
		*Z*_MgNa_^Mg^ = 4.5, *Z*_MgNa_^Na^ = 6, Δ*g*_MgNa_^0^ = 7,660.0 + 2.9*T*

hcp-(Mg)	Sublattice (Mg,Na)_2_(H,Va)_1_	^0^ *G*_Mg:H_^Mg_2_H^ = 173,217.6 − 242.672*T* + 2*G*(Mg_cph_) + 1/2*G*(H_2_, gas)
		^0^ *G*_Na:H_^Na_2_H^ = 2*G*(Na_cph_) + 1/2*G*(H_2_, gas)
		^0^ *G*_Mg:Va_^Mg_2_^ = 2*G*(Mg_cph_)
		^0^ *G*_Na:Va_^Na_2_^ = 2*G*(Na_cph_)
		^0^ *L*_Mg,Na::Va_^cph^ = 79,496 + 16.736*T*

bcc-(Na)	Sublattice (Na,Mg)_1_(H,Va)_3_	^0^ *G*_Na:H_^NaH_3_^ = *G*(Na_bcc_) + 3/2*G*(H_2_, gas)
		^0^ *G*_Na:H_^MgH_3_^ = *G*(Mg_bcc_) + 3/2*G*(H_2_, gas)
		^0^ *G*_Na:Va_^Na^ = *G*(Na_bcc_); ^0^ *G*_Mg:Va_^Mg^ = *G*(Mg_bcc_)
		^0^ *L*_Na::H,Va_^bcc^ = −5,569.8; ^1^ *L*_Na::H,Va_^bcc^ = −2,092.9
		^0^ *L*_Na,Mg:Va_^bcc^ = 30,000

MgH_2_	Stoichiometric	^Solid^ *G*_MgH_2__^0^ = −82,842.15 + 25.42*T* − 2.87*T*ln⁡*T* − 55.30 × 10^−3^ *T* ^2^ − 34,305.5*T* ^−1^
		298.15 ≤ *T* ≤ 2000

NaH	Stoichiometric	^Solid^ *G*_NaH_^0^ = −75,767.99 + 293.72*T* − 48.69*T*ln⁡*T* − 0.26 × 10^−3^ *T* ^2^ + 1.80 × 10^−8^ *T* ^3^ + 632,658.0*T* ^−1^
		298.15 ≤ *T* ≤ 2000

NaMgH_3_	Stoichiometric	^Solid^ *G*_NaMgH_3__ = −157,905.82 + 185.83*T* − 33.6*T*ln⁡*T* − 61.27 × 10^−3^ *T* ^2^
		298.15 ≤ *T* ≤ 2000

**Table 2:** Enthalpy and entropy of formation of MgH_2_.

Δ*H* (kJ/molH_2_)	Δ*S* (J/molH_2_ *·*K)	Temperature range (K)	Reference
−77.3	−136.9	298	This work
−77.4 ± 4	−138 ± 3	549–623	[44]
−74.4	−135	587–849	[35]
−70	−126	573–673	[48]
−78.2	—	573–616	[47]
−78.31	−140.07	513–633	[46]
−79	—	575–629	[45]
−66.9	—	450	[43]
−70.7	−119	590	[38]
−76.2	—	574	[94]
−74.05 ± 1.3	—	683	[32]

**Table 3:** Enthalpy of formation of the NaH phase.

Enthalpy of formation kJ/mole	Temperature K	Reference
−56.98	298	This work
−58.4 ± 1.2	623	[57, 78]
−56.9 ± 1.1	298	[76]
−56.44 ± 0.17	298	[75]

**Table 4:** Invariant reactions in the Mg-Na system.

Reaction	Temp./K	Composition, Na (at.%)			Reference
Gas → liquid 2	1153	92.5			This study
	1157	97.016			Modeling [86]

Liquid 1 → hcp-(Mg) + liquid 2	910	2.25	0.11	93.21	This study
	910	2.10	0.033	92.70	Modeling [86]
	911	2.0 ± 0.1			Experiment [81]
	911	2.1		98.6	Experiment [83]
	910	1.6		92.7	Experiment [82]

Liquid 2 → hcp-(Mg) + bcc-(Na)	371	99.97	1.0 × 10^−3^	100	This study
	371	99.98	4.15 × 10^−4^	100.00	Modeling [86]
	371				Experiment [81]
	371				Experiment [82]

**Table 5:** Thermodynamic properties of NaMgH_3_ decomposition from PCI and DSC experiments.

Reaction (2)		Reaction (3)		Δ_*f*_ *H* ^0^ (NaMgH_3_) (kJ/mol)	Reference
Δ*H* (kJ/molH_2_)	Δ*S* (J/molH_2_K)	Δ*H* (kJ/molH_2_)	Δ*S* (J/molH_2_ *·*K)		
85.45	127.2	114	154.2	−142.44	This work
—	—	—	—	−231∗∗	[13]
88 ± 0.9	—	—	—	−145∗	[18]
93.9 ± 6	116.2 ± 9	102.2 ± 4	125.9 ± 6	−145∗	[15]
94 ± 15	140 ± 22	116 ± 2	165 ± 3	−152∗	[17]
92	123	—	—	—	[27]
86.6 ± 1	132.2 ± 1.3	117	168.2	−145.1∗	[88]

^*^The values are (re)calculated in this work using Δ*H* values reported in the literature; ∗∗data obtained by DSC measurements.
